# Investigating preferences for color-shape combinations with gaze driven optimization method based on evolutionary algorithms

**DOI:** 10.3389/fpsyg.2013.00926

**Published:** 2013-12-13

**Authors:** Tim Holmes, Johannes M. Zanker

**Affiliations:** ^1^Acuity Intelligence Ltd.Reading, UK; ^2^Department of Psychology, Royal Holloway University of LondonLondon, UK

**Keywords:** visual perception, aesthetics, shape, color, evolutionary algorithm, individual preference

## Abstract

Studying aesthetic preference is notoriously difficult because it targets individual experience. Eye movements provide a rich source of behavioral measures that directly reflect subjective choice. To determine individual preferences for simple composition rules we here use fixation duration as the fitness measure in a Gaze Driven Evolutionary Algorithm (GDEA), which has been demonstrated as a tool to identify aesthetic preferences (Holmes and Zanker, 2012). In the present study, the GDEA was used to investigate the preferred combination of color and shape which have been promoted in the Bauhaus arts school. We used the same three shapes (square, circle, triangle) used by Kandinsky (1923), with the three color palette from the original experiment (A), an extended seven color palette (B), and eight different shape orientation (C). Participants were instructed to look for their preferred circle, triangle or square in displays with eight stimuli of different shapes, colors and rotations, in an attempt to test for a strong preference for red squares, yellow triangles and blue circles in such an unbiased experimental design and with an extended set of possible combinations. We Tested six participants extensively on the different conditions and found consistent preferences for color-shape combinations for individuals, but little evidence at the group level for clear color/shape preference consistent with Kandinsky's claims, apart from some weak link between yellow and triangles. Our findings suggest substantial inter-individual differences in the presence of stable individual associations of color and shapes, but also that these associations are robust within a single individual. These individual differences go some way toward challenging the claims of the universal preference for color/shape combinations proposed by Kandinsky, but also indicate that a much larger sample size would be needed to confidently reject that hypothesis. Moreover, these experiments highlight the vast potential of the GDEA methodology in experimental aesthetics and beyond.

## Introduction

For many centuries questions about the origin, rational, universality, and biological foundations of aesthetic judgments have been a matter of speculation and debate. Since Fechner's inception of “psychophysics” as an exact method of studying the relationship between the physical world and mental experience (Fechner, 1876) such questions have been within the reach of experimental investigation. Berlyne's work (1971) provides a rich foundation to advance this experimental approach by applying recent technical and computational advances in psychophysics that allow us to collect and analyse large and complex data sets in an attempt to revisit some of the classic questions of aesthetics. In the present paper we wanted to test experimentally a well known composition rule about the association of color and shape, which had been advanced in the Bauhaus arts school by one of its most prominent members, Wassily Kandinsky. His proposal that red squares, yellow triangle, and blue circles are the “most pleasing” became a prominent topic at the Bauhaus art school teaching and has been tested empirically with mixed results (Makin and Wuerger, 2013).

The effects of color, shape, and space were one of the primary concerns of the Bauhaus art school (Wingler et al., 1969). As a painter and a teacher, Kandinsky was an influential member of the school. In addition to that, he was a self-professed synaesthete (Ione and Tyler, 2003; Kadosh and Henik, 2007), and had a profound interest in the combination of such features as color and shape in a single object. In addition to his associations between color and music, Kandinsky was convinced that there are universal harmonies between shape and color. In particular, he claimed that there were strong associations between the primary colors blue, red, and yellow and simple geometric shapes like circles, squares, and triangles (Jacobsen, 2002). He drew support for this claim from an empirical investigation of this association by presenting students with a questionnaire containing the outline of a circle, a square, and a triangle and asking them to color in the shapes using only red, blue, and yellow, with each color to be used once and only once (Kandinsky, 1923). The experiment suggested a group preference for blue circles, red squares, and yellow triangles (Jacobsen, 2002). The reliability of this study is contentious (Ball and Ruben, 2004), since Kandinsky's own students and colleagues were unlikely to provide an unbiased sample, and the precise distribution of the results is, unfortunately, undocumented. Additionally the strict color limitations and pre-printed questionnaire which fixed both the arrangement and orientation of the three shapes did not allow the associations to vary independently or for order and orientation effects to be explored. Unsurprisingly, subsequent attempts to validate the theory have yielded inconsistent results (Jacobsen, 2002; Leder et al., 2004; Albertazzi et al., 2013), perhaps because they have tended only to try to establish group level associations, and mostly within the strict confines of Kandinsky's original questionnaire without allowing participants to freely explore a broader color space [with the notable exception of Albertazzi et al. (2013)]. Moreover, several replications have suggested systematic differences between the color/shape associations at an individual level without devoting much effort to investigating the strength and consistency of color/shape correspondence on an individual level (Jacobsen, 2002).

In some of our recent work (Holmes and Zanker, 2008, 2012) we established a novel method to investigate individual choices in large search spaces, based on a gaze driven evolutionary algorithm (GDEA), which has the potential to address some of the issues that had been raised with Kandinsky's experiment. This method combines the power of evolutionary algorithms (EA)—a computational optimization method for highly multi-dimensional search spaces—with the versatility of gaze tracking as means to collect participants' responses in an unobtrusive way from the natural behavioral responses to evaluating the stimuli without focusing the participant on the question being explored. The GDEA was used in our experiments to investigate color/shape association on both a group and individual level. Participants were presented with the same three shapes as used by Kandinsky, but with both the three color palette offered in the original experiment as well as an extended palette of seven colors. In addition, the effects of shape orientation were also explored. The limitation of singular color-shape combinations was overcome, as well as possible position or sequencing effects.

**Figure 1:**
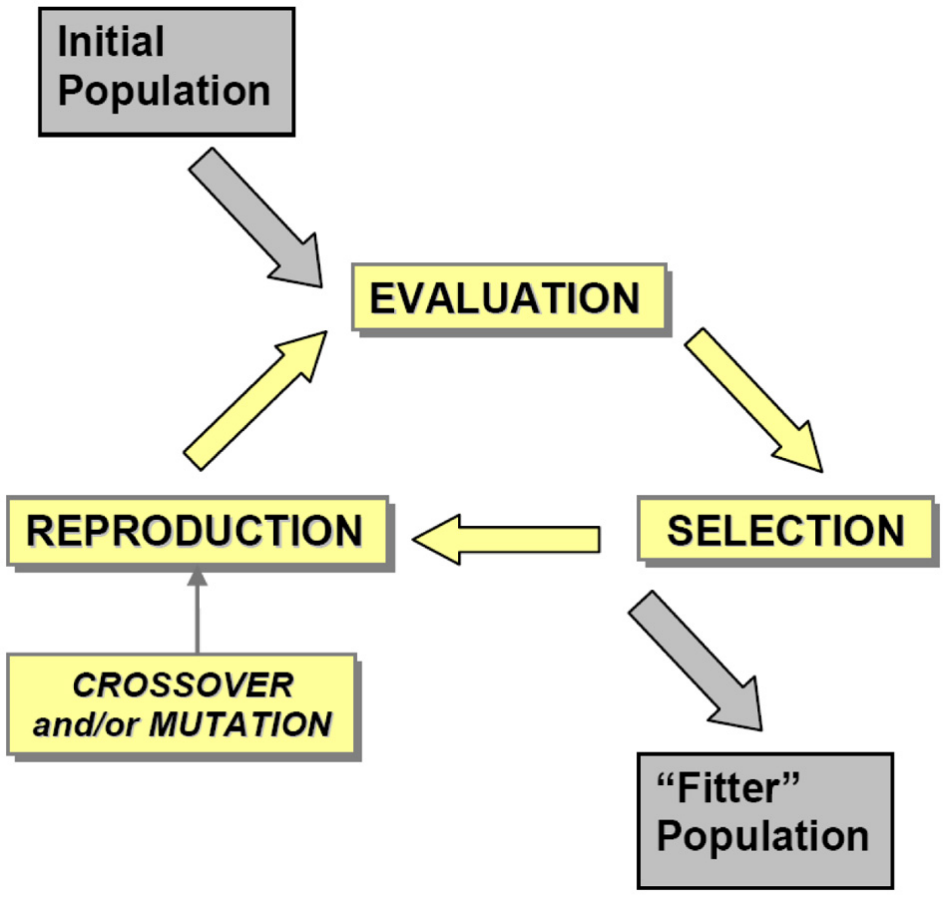
**The evolutionary cycle.** An initial population, usually randomly generated, is evolved over several generations using an iterative process comprising EVALUATION, SELECTION and REPRODUCTION stages to produce a “fitter” population—the average fitness of the overall population has increased. EVALUATION is the means by which each individual in the population is attributed a fitness score, which is using gaze data in the GDEA. SELECTION is the means by which individuals are chosen to participate in the generation of “offspring” which will populate the next generation, and thus carry forward the genetic information contained in the chromosomes of both of the parents. REPRODUCTION is the means by which the genetic information from the “parents” is recombined to produce offspring, typically involving crossover (an exchange of genetic information from both parents, resulting in inheritance of properties from both parents) and mutation [random changes to the chromosome of the offspring resulting in novel properties not inherited from the parent(s)].

EA are an optimization method which have, at their core, the biological evolutionary principles of reproduction and selection applied to a large population of individuals over a number of generations. Individual members of a population represent possible solutions to a design problem and are described by genetic information using a chromosome which can be thought of as a multi-dimensional vector, and reproduce by exchanging parts of this genetic code with that of another individual (crossover) to produce “offspring” with chromosomes that inherit information from both “parents.” Random mutation is often applied to the genetic codes of the offspring to introduce new variants into the population. Survival of offspring, and ability to pass genetic information onto a subsequent generation, is determined by an evaluation and selection process which ensures the fittest members of the population are most likely to reproduce and send their genetic codes on to the next generation (Holland, 1975; Goldberg, 1989; Bentley, 1999). An “evolutionary run” comprises of repeated iterations of this process, as shown in Figure 1, which at the end converges to a “final generation” in which the population represents the evolved state that is regarded as the best solution for a given design problem.

These concepts from biological evolution engendered several different types of EA which were developed independently (Eiben and Smith, 2003), which incorporate different genetic representations, such as binary encoded genes in Genetic Algorithms (GA) (Holland, 1975; Goldberg, 1989), real-valued genes in Evolutionary Strategies (ES) (Rechenberg, 1973, as cited in Rechenberg, 1989; Schwefel, 1993), or logical expressions in Genetic Programming (GP) (Koza, 1992). Furthermore, they can rely to different extents on the variance from recombination and mutation (Spears, 2000). In the present context, we use a hybrid mechanism that uses integer values for genes (similar to ES) and uses crossover as well as mutation to boost genetic variability, which puts it into the category of a Genetic Algorithm. One of the crucial technical aspects of the GDEA method used here concerns the implementation of the selection. In search of preference an active choice paradigm has been previously used (see Holmes, 2010) which requires the participants to press buttons in order to indicate their choices, or make verbal responses, which leads to very time-consuming experiments, and corresponding challenges for maintaining attention, and to tendencies to think extensively about every decision, which could trigger criteria shifts about what is regarded as “best,” “pleasing,” or “beautiful.”

Therefore, the GDEA makes use of spontaneous eye movements (Holmes and Zanker, 2012) which result from an interaction between so called “bottom-up” (e.g., saliency) and “top-down” (e.g., attractiveness) effects which can be used as a largely reflexive physiological marker for preference. Preferential selection based on eye movements has been used in a variety of contexts, in particular being at the core of the preferential looking paradigm (Teller, 1979; Dobson, 1983). The power of such methods has been supported by the gaze cascade model of decision making based on preference in visual displays (Shimojo et al., 2003; Glaholt and Reingold, 2009). The relationship between accumulated fixation time and task relevance, which is fundamental to such methods, has first identified by Yarbus (1967).

The purpose of the current work is to explore whether preferred associations of color and shape can be confirmed with newly developed experimental techniques that reduce the influence of potential bias in the task imposed on participants, and also allow us to test such basic aesthetic preferences in a less constrained set of stimuli. To this end we use a specific version of the GDEA that allows us to evolve preferred color-shape combinations for the three basic shapes used by Kandinsky in the absence of instructions that might lead the participants, with simultaneous presentation and comparison of design alternatives, with a larger color palette, and allowing shapes to be presented in non-canonical (upright) orientation. All of these manipulations would lend support to any claim of a “general” composition rule that goes beyond the very small set of designs that would have been possible in Kandinsky's original experiment.

## Methods

The basic structure of an evolutionary algorithm is well defined through the fundamental steps of reproduction and variation combined with evaluation and selection (see Figure 1). However, the implementation of various processes, both in terms of logic and in terms of specific runtime parameters, varies considerably with each instantiation of an evolutionary algorithm. In the following section we describe the representation of features in the genetic code, and the selection, reproduction, and mutation processes used in this study, as well as the subjective fitness measure driving the selection process.

### Genetic code to represent stimuli

The phenotype were defined as colored shapes which could be displayed in any of eight orientations. This was represented using a single chromosome containing three genes as follows:

- Shape Gene—1 = circle, 2 = triangle, 3 = square.
- Color Gene—1 = red, 2 = orange, 3 = yellow, 4 = green, 5 = cyan, 6 = blue, 7 = magenta.
- Clockwise Rotation Gene—1 = 0°, 2 = 45°, 3 = 90°, 4 = 135°, 5 = 180°, 6 = 225°, 7 = 270°, 8 = 325°.

Integers were used to encode each gene with three, seven, or three different values (alleles), respectively. Thus, the chromosome simply comprised three integers, giving a relatively small solution space of just 168 possible genotypes. It should be noted that due to the rotational symmetry properties of squares and circles, this leads to a considerably smaller number of phenotypes (47).

### Evolutionary algorithm

When using binary genes in GA, mutation can be implemented using a random bit-flip which rarely need constrained on the number of bit-flips which could occur in a single offspring. For features determined by several or multi-graded genes such an implementation can result in mutated individuals which can look very different from their parents since the probability of any one bit (allele) in chromosome being mutated is the same. This potentially disrupts the function of the mutation operator which is primarily to perform a localized movement within the search space (Eiben and Smith, 2003). In the present experiment a form of geometric mutation (Moraglio and Togelius, 2007) was applied, which is a proximity weighted mutation operator. This means that the probability of any one allele mutating to a neighboring value is higher than the probability of it mutating to a distant one. In the present context a single mutation in any of the genes resulted in a change to one of its two nearest neighbors, each of which was equally likely, which would generate the smallest deviation within a feature, such us the most similar orientation or color of an object. The mutation for each given gene was determined independently by means of a “weighted coin toss” (i.e., true/false values are not equally likely), meaning that it was possible for multiple mutations within the same chromosome to occur, such as shape and color changing in a single evolution step.

We used a tournament selection method, which has been suggested for situations where the fitness scores are noisy, as is typically the case in interactive evolutionary computation (Miller and Goldberg, 1995; Takagi, 2008) where humans provide the fitness scores rather than mathematical functions whose results are determined by the alleles in the chromosome. Tournament selection ensures that the fittest population members are most likely to be selected as parents in the reproduction process whilst still allowing weaker members to contribute to the variability of the population. In this case, for each offspring, tournaments of three randomly selected population members were created from which the two fittest members were chosen to act as parents.

A single evolutionary run comprised 10 generations, with two presentations of eight individuals for each generation. A steady state (fixed) population size of 36 members was used which was sufficient due to the relatively small solution space. The initial population was generated by using random genomes and subsequent generations were evolved with crossover rate of 0.75, and a mutation rate of 0.1875 together with partial replacement, i.e., replacing a fraction of the genes from the parent population (75%), was used to preserve diversity across consecutive generations and to limit premature convergence. The mutation rate is relatively high for a GA where rates of 0.05 are more common, but within the small solution space we did not want participants losing interest as a result of seeing screen after screen of the same shape/color combinations.

### Stimuli

Samples of eight individuals (colored shapes) were rendered and displayed, using a Cambridge Research Systems ViSaGe (Visual Stimulus Generator), on a 48 cm diameter CRT Monitor (Sony 17′ Multiscan 17SF 1280 × 1024 pix) at a distance of 57 cm from the participant. All experimental software was written in Matlab R2007b, using a Windows XP system. In Kandinsky's original questionnaire, the area of the three shapes was not kept constant, but instead the height and width were. Because a constant area would result in triangles that were perceptually larger than the other shapes, we also used constant width and height (32 mm) resulting in areas of 805 mm^2^ for a circle, 512 mm^2^ for a triangle, and 1024 mm^2^ for a square. We presented eight (genotypically) distinct individuals on each screen, but the same individual could be displayed more than once in conditions using more than one screen presentation for fitness evaluation. It should also be noted that due to the relatively small solution space, multiple individuals with same phenotype frequently occurred in a single presentation. Individuals were displayed in a radial fashion, with their center at 8° distance from the center of the screen. Thus, the stimuli were presented peripherally with respect to the central fixation cross-hair presented immediately prior to the stimuli. A white background was used and all individuals were rendered with a black outline (2 pixels wide), in order to approximate the white paper and black outlined shape to be colored in from the original questionnaire used by Kandinsky (1923). Luminance values for seven different colors used to render the shapes on the screen were: red (RGB = 255|0|0, 18.0 cd/m2; CIE XYZ = 41.24, 21.26, 1.93), orange (RGB = 255|128|0, 47.4 cd/m2; CIE XYZ = 48.96, 36.70, 4.50), yellow (RGB = 255|255|0, 76.9 cd/m2; CIE XYZ = 77.00, 92.78, 13.85), green (RGB = 0|255|0, 58.9 cd/m2; CIE XYZ 35.76, 71.52, 11.92), cyan (RGB = 0|255|255, 63.6 cd/m2; CIE XYZ 53.81, 78.74,106.97), blue (RGB = 0|0|255, 9.5 cd/m2; CIE XYZ 18.05, 7.22, 95.05), magenta (RGB = 255|0|255, 22.7 cd/m2; CIE XYZ 59.29, 28.48, 96.98). Background luminance (white) was (RGB = 255|255|255, 86.3 cd/m2; CIE XYZ 95.05, 100.00, 108.90).

**Figure 2:**
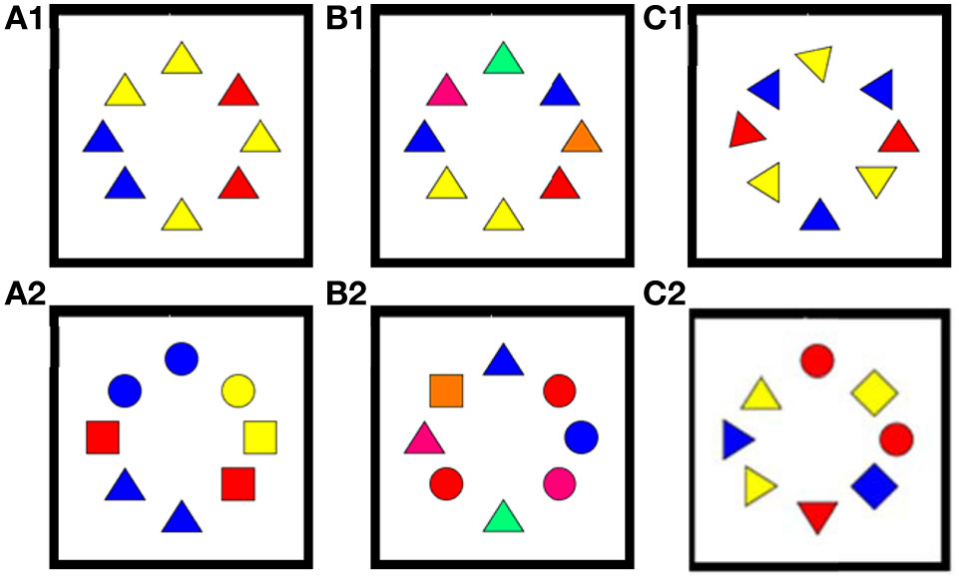
**Example stimuli for the six conditions used.** The first two conditions **(A,B)** did not include any manipulation of orientation. **(A1)** A screen from the single shape, three colors condition—other trials featured eight circles or eight squares. **(A2)** A screen from the three shapes, three colors condition. **(B1)** A screen from the single shape, seven colors condition—other trials featured eight circles or eight squares. **(B2)** A screen from the three shapes, seven colors condition. For the two conditions A and B the rotation gene was implemented. **(C1)** A screen from the single shape, three colors, eight orientations condition—other trials featured eight circles or eight squares. Note that the eight alleles of the orientation gene result in only one phenotype for the circle and two phenotypes for the square due to the degree of symmetry in those shapes. Shows a screen from the three shape, three colors, eight orientations condition.

Participants were presented with samples of eight colored shapes and instructed to look for the most aesthetically pleasing shape, the specific target, circle, square, or triangle being indicated via an onscreen instruction at the start of each 10-generation run. In all, eight conditions were presented, six of which are shown in Figure 2.

(A1) Single shape, 3 colors, no rotation (3 phenotypes)
(A2) Mixed shapes. 3 colors, no rotation (9 phenotypes)
(B1) Single shape, 7 colors, no rotation (7 phenotypes)
(B2) Mixed shapes. 7 colors, no rotation (21 phenotypes)
(C1) Single shape, 3 colors, 8 rotations (24 phenotypes)
(C2) Mixed shape, 3 colors, 8 rotations (72 phenotypes)

Two additional condition, in which eight different rotations were used for singe and mixed shapes, respectively, with the seven color palette were also tested in our experiments (as conditions D1 and D2), but the data are not shown in this paper because they only added little to the observations made in the set presented here (for a full account, see Holmes, 2010). The experiment was run in two parts. Conditions A and B, in which the rotation gene was ignored during phenotype rendering, was completed by six participants with 12 evolutionary runs for each condition. Conditions C (and D) with the rotation gene activated were completed by three participants with 12 evolutionary runs for each condition. An underlying population size of 36 individuals was used for all conditions.

### Procedures

Participants were presented with samples of eight colored shapes and instructed to look for the most aesthetically pleasing shape, the specific target, circle, square or triangle being indicated via an onscreen instruction at the start of each 10-generation run.

Participants were initially presented with a white screen with a central fixation cross-hair for 1000 ms. Samples of eight individuals from the population were then presented together for 1500 ms after which a color noise mask (randomly generated 5 × 5 pixel blocks, 2.5 × 2.5 mm, of red, orange, yellow, green, cyan, blue, and magenta, see Holmes (2010) was presented for 250 ms to neutralize retinal after images from the high contrast stimuli before the fixation cross was presented to start the next iteration.

### Eye-tracking and fitness estimation

A Cambridge Research Systems 50 Hz Video Eye-Tracker (CRS VET) was used with CRS Matlab toolbox. Fixations were defined as periods of 100 ms or more during which the gaze location remained within a 2.5 × 2.5 mm window on the screen i.e., within a 0.25° region of visual angle at a viewing distance of 57 cm.

After presentation of each screen, the gaze data was analyzed as follows, in order to derive an eye tracking signature that was simply cumulative fixation time. (i) Any positional information which did not fulfill the criteria for a fixation was removed as well as any fixation which lay outside of a non-overlapping area of interest extending 2.5 mm beyond the perimeter of each individual rectangle (phenotype). (ii) The total amount of time spent fixating in each zone was then calculated and divided by the screen presentation time to give a fitness score for each phenotype in the range 0.0–1.0. (iii) In the cases where an individual was presented on multiple screens in a single generation, the fitness scores for each presentation were averaged to produce a single fitness score for the phenotype. It is important to note that all fixations for the zone enclosing the entire phenotype contribute to the fitness of that phenotype; fixations on individual features within the phenotype were not distinguished meaning that the fitness is truly based on the interaction of genes resulting in the phenotype and not the individual genes themselves, as is typical for most EA (Takagi, 2001).

Because the number of individuals presented to the participant was less than the size of the population, the fitness of individuals in the population that had not been presented for evaluation was estimated prior the reproduction step, using the following procedure. Let for any one generation *x*_*i*_ represent the *i*-th population member, with an associated fitness Č(*x*_*i*_) defined as the average amount of time spent fixating on that individual. Then

$$f(xi)=T(xi)/N(xi)\text{​}/\text{​}\sum_{i}T(xi)/N(xi)$$

Where *T*(*x*_*i*_) is the total amount of time spent fixating on the *i*-th population member, and *N*(*x*_*i*_) is the total number of presentations of the *i*-th population member.

Once all samples had been presented for one generation, the fitness of each un-presented individual was estimated. Let *H*(*x*_*i*_, x_*k*_) be the “Euclidian distance” between the *i*-th (un-presented) individual and the *k*-th (presented) individual. This is calculated for *x*_*i*_ for all un-presented *x*_*k*_, and the method of least squares is then used to estimate the equation of the line which best describes the points [*H*(*x*_*i*_, *x*_*k*_), *f*(*x*_*k*_)], which can then be used to estimate the fitness of the un-presented individual as follows:

$$\hat{f}(xj)\;=\;\alpha H(xj,xj)+\beta$$

Since the Euclidian distance of an individual with itself is always zero, β gives the estimated fitness of the un-presented *l*-th population member. This process was repeated for all un-presented population members, *i*, before the selection and reproduction stages of the algorithm.

### Participants

Six participants were recruited from the Psychology Department of Royal Holloway University of London, and received no payment for completion of the experiment. The experiments comply with general ethical procedures, and had been approved by the local ethics committee. All participants conformed that they had normal or corrected-to-normal vision, and no color deficiency, and provided written consent.

## Results

### Group results

Similar to our previous work on aesthetic preferences, such as our study on the Golden Ratio (Holmes and Zanker, 2008), choices of individual participants could vary considerably suggesting that a much larger sample would be needed to collect evidence about the existence of systematic effects and to discern their significance with sufficient statistical power. However, the results even from the small group of participants tested in the current experiment do suggest some degree of individual color/shape correspondence. Throughout this section, stacked bar charts are used to illustrate the average proportions of particular colors for a given shapes and orientation, and at any given generation in the evolutionary run, which are found as phenotypes in the stimulus population. In the presence of any selection pressure that would favor a particular design, these proportions would deviate from random populated samples of feature combinations. For example in the single shape, single orientation, three color condition (A1) and the population size of 36 individuals, the solution space has the size three (colors) for each given shape, which should lead to 1/3 (33%) of observations or 36/3 = 12 individuals for each phenotype (here color) in an not evolved population (such as the initial, random selected, generation). In the course of evolutionary change, these proportions would change, and the population would contain a larger proportion of the “preferred” phenotype, whilst the proportion of other phenotypes would decrease correspondingly.

**Figure 3:**
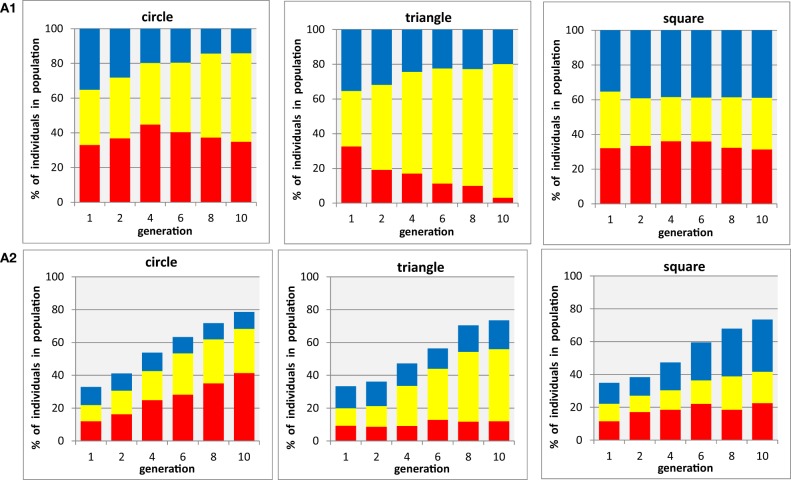
**Evolution of populations with three shapes × three colors. (A1)** three shapes are tested in separate populations containing a single shape each (i.e., participants are asked to select the preferred individual from three color-shape combinations with identical shape). **(A2)** three shapes are tested in mixed population containing all shapes (i.e., participants select the preferred color-shape combination of a given shape in the presence of both other shapes, combined with the same three colors: nine color-shape combinations). Each panel shows for one particular shape (circle, triangle, square) the development of color proportions of red, yellow, and blue individuals (shown as stacked bars) in an evolutionary run, displayed for generations 1, 2, 4, 6, 8, and 10 (abscissa). Because the mixed shape population in **(A2)** does contain other shapes as well, the proportions for each individual shape do not add up to 100%. Averages from *n* = 6 participants.

The three upper panels illustrating condition A1 in Figure 3 shows the proportion of red, blue, and yellow individuals in a population that only contains circles, triangles, or squares, respectively. These are shown for the initial (randomly selected) generation 1, and generations 2, 4, 6, 8, and 10, which were evolved using the methods described in section Methods. As expected, in the first generation for each shape the populations appear to be well balanced with about equal proportions of all three colors. There is a slight trend for yellow circles to increase in proportion and for blue circles to decrease, and a much more prominent one for yellow triangles to increase at the expense of red, whilst for squares there is no consistent trend. The three lower panels illustrating condition A2 in Figure 3 show the proportion of red, blue, and yellow individuals of one particular shape in a population that contains mixtures of circles, triangles, and squares that can take any of the three colors, for generations 1, 2, 4, 6, 8, and 10, as in A1. The first observation to make for this condition is that the initial generation not only shows equal proportions of all three colors (as in A1) but additionally is reduced to a cumulative proportion for all colors of ~33% for each of the three shapes—again as expected, because the rest of the randomly generated population should be made up of the two other shapes. The overall proportion of the selected shape grows from this level 70–80% in generation 10 for each of the shapes, as a result of participants complying with the task to choose their preferred color combinations for this particular shape, at the expense of reducing the proportion of alternative shapes of any color. It is important to note that even at the end of the evolutionary run the population does not reach a 100% level of the selected shape because the genes for other shapes are not completely eliminated from the population, although it is clear that other shapes are substantially reduced (i.e., the participants complied with the task, looking for the right shape). Within each of the shape panels for condition A2, we find similar but not identical trends to those observed made in condition A1: a substantial growth of yellow and red for circles, a considerable increase in yellow for triangles, a substantial increase of blue and modest increase of red in squares.

In general, it is interesting to observe that the presentation of mixed shapes results in clearer, and more distinct preferences for each of the three shapes (red circles, yellow triangles, and blue squares) than it was the case when the shapes were presented in isolation, suggesting that the mixed condition perhaps best replicates Kandinsky's experiment since participants seem to have associated a single color and shape. In two additional experiments, during which each shape was either presented in isolation or containing a mixture of all three shapes throughout the generations, with different search spaces were designed to find out whether such a trend can be generalized across a range of conditions.

**Figure 4:**
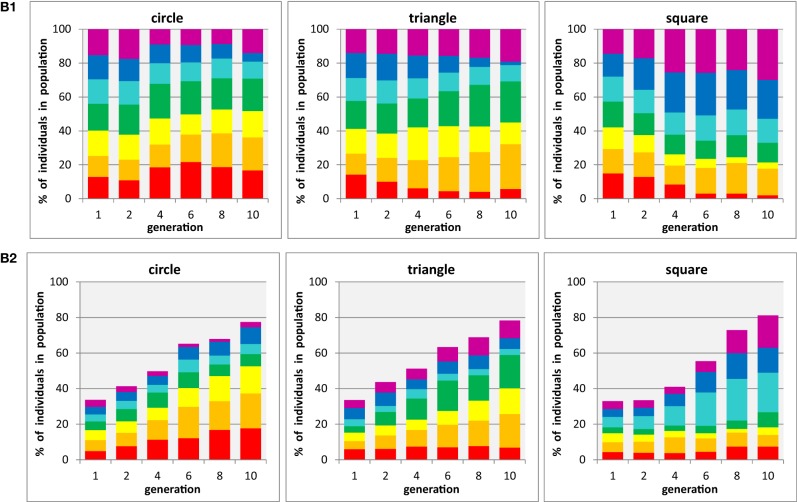
**Evolution of populations with three shapes × seven colors. (B1)** three shapes are tested in separate populations containing a single shape each (i.e., participants are asked to select the preferred individual from seven color-shape combinations with identical shape). **(B2)** three shapes are tested in mixed populations containing all shapes (i.e., participants select the preferred color-shape combination of a given shape in the presence of both other shapes, combined with the same set of seven colors: 21 color-shape combinations). Each panel shows for one particular shape (circle, triangle, square) the development of color proportions of red, orange, yellow, green, cyan, blue, and magenta individuals (shown as stacked bars) in an evolutionary run, displayed for generations 1, 2, 4, 6, 8, and 10 (abscissa). Averages from *n* = 6 participants.

So far, our experiments were restricted to the three “primary” colors used by Kandinsky in his work, red, yellow, and blue, which does not speak to any preference outside this very restricted range of choices, on the presence of other potential candidates. The strength of our current experimental method is to probe large “solution spaces,” i.e., preferred coloration from a larger color palette. The results of two tests, during one of which each shape was presented in isolation, and the other containing a mixture of all three shapes throughout the generations, are summarized in panels B1 and B2, respectively, of Figure 4.

The first impression of the overall figure, being very colorful, seems suggest that there is not a dominating subset of preferred color, but a rather mixed set of choices that can develop o preference for certain combinations over time. For instance in the left two panels, for circles, one can see a weak preference for red developing that mirrors that observed in Figure 3 for the minimal color palette, but there is no similarity for yellow preference. It should be noted, however, that for a set of colors defined by yellow and its two neighbors (orange on green) will occupy more than half of the final population and that the two colors furthest away from red and yellow (blue and cyan) end up with smallest probabilities in the single shape condition, whereas in the mixed shape condition a strong dominance for a yellow-orange-red group develops, Similarly, the final population of triangles is dominated by yellow and its two neighbors, orange and green in both conditions, and squares leading to a cyan-blue-purple dominance that in a wider sense reflect the preferences in the restricted color palette. In conclusion, some level of color preferences do develop in both experiments (A and B), but the preference seems not to be narrowly restricted to a particular shade of colors (which could relate to Kandinky's initial thoughts about color and angles, see Kandinsky, 1926), but rather link to a broad range of similar colors on the color circle.

**Figure 5:**
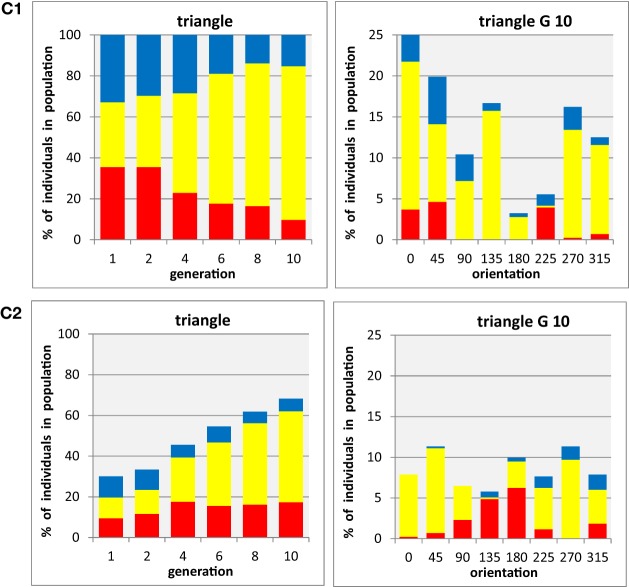
**Evolution of populations with three shapes × three colors × eight orientations. (C1)** shapes are tested in separate populations containing a single shape each, data shown for triangle (i.e., participants are asked to select the preferred triangle from 24 color-orientation combinations). **(C2)** all three shapes are tested in mixed population containing all shapes (i.e., participants select the preferred color-shape combination of a given shape presented at any one of eight orientations in the presence of both other shapes, combined with the same set of colors and orientations: 72 color-shape-orientation combinations). The panels on the left side shows for triangles the development of color proportions of red, yellow, and blue individuals (shown as stacked bars), averaged for all orientations, in an evolutionary run, displayed for generations 1, 2, 4, 6, 8, and 10 (abscissa). The panels on the right side show the proportions of red, yellow, and blue individuals in the final (10th) generation in an evolutionary run, for each of eight stimulus orientations (abscissa). Averages from *n* = 3 participants.

Whereas experiment B1/B2 allowed us to expand the observations of preferred colors beyond the restricted set of three primary colors used by Kandinsky, we can also ask a corresponding question about the constraints about the shapes that have been associated with colors. Still keeping the limited set of three every basic shapes used by Kandinsky, we made an initial step of expanding the search space in this respect by a further set of evolutionary runs (with three participants) allowing different orientations of these shapes with respect to the vertical. For instance, the Apex of the triangle could be rotated such that they point in eight different directions (cf. Figure 2), thus increasing the large “solution spaces” considerably for this configuration. Because corresponding rotations only lead to two distinct appearances of squares (orthogonal and oblique squares, respectively) and identical phenotypes for circles, due to their specific properties with respect to rotation symmetry, we focus here in the results for triangles (which in our preceding experiments also showed the strongest baseline color-shape association). The results of two tests for this shape, during one of which each shape was presented in isolation, and the other containing a mixture of all three shapes throughout the generations, are summarized in panels C1 and C2, respectively, of Figure 5.

The two panels in the left side of Figure 5 illustrate the time course of evolution in the present of an orientation gene, showing the average proportions of the three colors in triangles, irrespective of the orientation of the shape chosen by the participants. The striking similarity of these two diagrams with those shown in Figure 3 for triangles (the middle panels in A1 and A2) clearly demonstrates that the additional freedom to select an orientation did not change the color preferences associated with the three basic shapes. The two panels on the right side of Figure 5 show the proportions of colors associated with each triangle orientation in the final generation of the evolutionary runs for each of the eight orientations separately. Because the overall number of individuals was split into eight subsets for this analysis (note the different scaling of on the ordinates), the data are much noisier than those in the previous figures. There might be a weak tendency of overall preference for upright orientations (compare stacked bar sizes around 0° with those around 180°), and as result of this the preference for yellow seems to be more pronounced in these orientations. However, there is no clear and systematic association of any particular color with any particular orientation of the triangles, suggesting that the preferred association of yellow with triangles is a genuine property of the shape rather than being related to its particular orientation.

### Variability between single participant

**Figure 6:**
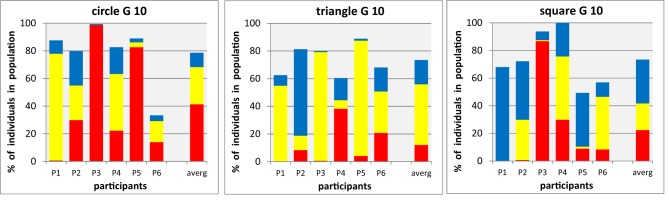
**Final stage of mixed shape populations with three shapes × threecolors (individual data from A2 for *n* = 6 participants, P1–P6, for details see Figure 3).** Each panel shows for one particular shape (circle, triangle, square) the proportions of red, yellow, and blue shapes (shown as stacked bars, ordinate) in the final generation (10) of the evolutionary runs, displayed for six participants (abscissa) and for their average (stacked bar at the right of each panel).

It is an important question whether the existence or absence of preferences for particular shape-color combinations, and the strength of any preference, in the color proportions at group level shown in section Group Results, is the result of individual variations (i.e., different but pronounced color preferences for different participants) or a property of the association between color and shape itself (i.e., variability of individual decisions, or gene frequencies within the same participant). It is important to keep in mind that an inherent, and crucial, feature of the genetic algorithm itself is to sustain variability in the population by selection and mutation. Whilst this means that we have to expect genetic diversity in any population generated by an individual participant in our experiments, inter-individual differences in color proportions would still be reflected by convergence toward different color proportions for different individuals. This question will be followed up by looking at some individual date from the same experiments in more detail. In Figure 6 we show for each of the three shapes (shown in separate panels) the proportions of colors in condition A2 (three colors, mixed shapes) in the final population, separately for each of the six participants (columns with labels), together with the group averages (column seven at the right of each panel).

The amount of inter-individual variability is immediately apparent when looking at any of the data panels: for instance, for circles, on participant expresses a strong preference for yellow, two others for red, and the three remaining participants end up with a range of colors in their final populations. There is also considerable variation of the proportion of other shapes which remaining in the final population, as expressed by the overall height of the stacked bar—in participant P6, more than 60% of the final population is made up from triangles and squares, despite the instruction to participants to look for their preferred circles. Comparing the results from different shapes, we find participants with distinct color-shape associations (such as P5 with red-yellow-blue dominance for circle-triangle-square) others with preferred colors (such as P3 with red-yellow-red, or P1 with yellow-yellow, blue), and others with very little distinct preferences (such as P4)

**Figure 7:**
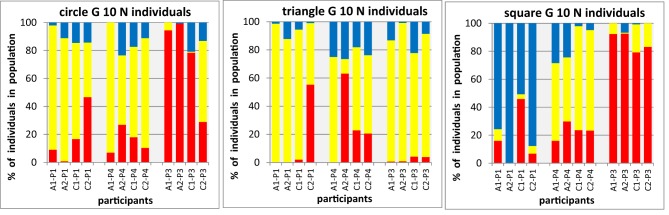
**Color proportions in final populations for three participants in four different conditions (as shown as averages in A1, A2 and C1, C2 in Figures 3, 5).** Each panel shows for one particular shape (circle, triangle, square) the proportions of red, yellow, and blue population members (shown as stacked bars, ordinate, to facilitate the comparison across conditions all data are shown here normalized as percentages of a particular shape in each of the three colors) in the final generation of the evolutionary runs, displayed for each of the three participants (P1, P4, P3) as a block of four condition (abscissa).

Observing in Figure 6 distinct, but not identical, references for a particular form-shape associations in some participants raises another interesting question, about the internal consistency of variations: is an individual preference a reproducible association that would be conserved over extended periods of time or just a spontaneous preference arbitrarily expressed at the moment in time when this experiment was carried out? A first answer to this question arises from the comparison of experimental data of participants across different experiments that could have been separated by many weeks. Three of our participants were tested in all of the experimental conditions reported here, and therefore offer a good data set for such a comparison, which we restrict to the conditions involving the combination of three colors and three shapes that are straight forward to compare. In Figure 7 we show for each of the three shapes (shown in separate panels) the normalized proportions of colors in condition A1, A2 (individual, mixed shapes, upright orientation) and C1, C2 (individual, mixed shapes, pooled across eight directions) in the final population, separately for the three participants (columns for four conditions shown in a block for each participant).

A general inspection of Figure 7 gives a clear impression of color patterns in the bars, which varies considerably between different panels (i.e., shapes) and between different participants (blocks within each panel), but exhibit an impressive resemblance with each other for a give participant and condition (within a block of bars), For example, for squares (right panel of Figure 7) in each of the respective bars there is a clear dominance of blue for participant P1, of yellow for participant P4, and red for participant P3. This suggests a considerable persistent of individual color preferences for shapes (i.e., low intra-individual variation) in the presence of substantial differences of color preferences between individuals (i.e., high inter-individual variation).

## Discussion

GA are commonly used in engineering applications as an optimization tool and have been explored in our previous work as a powerful method for studying human decision making, by using the subjective responses of human observers to visual stimuli to evolve a preferred stimulus. In particular, in our initial work (Holmes and Zanker, 2008) conventional key press responses for the selection of the preferred designs, in that case aspect ratios of simple rectangles, was replaced by selection based on the amount of time spent fixating the individual rectangles which was recorded using an eye-tracker. This method was expanded by subsequent experiments (Holmes and Zanker, 2012) which developed an oculo-motor signature based on several aspects of eye movements toward and between targets which provide a more specific reflection of choice, and was tested with a wider range of stimuli rich in a number of image attributes, supporting optimization across much larger solution spaces. An additional advantage, which is crucial for the current study, arises from the possibility to investigate choice preferences at the individual level rather that being restricted to choice frequencies determined for groups of observers.

The present work uses this technique to generate profiles of individual and group preferences and confirms that the GDEA methodology seems sound when extending beyond single gene and monochromatic phenotypes, which is clearly an important step when using it to evaluate questions of aesthetics. It is clear from Figures 3–5 that the Evolutionary algorithm develops smoothly and consistently from random choices (i.e., balanced proportions of colors) in generation one through consecutive generations to characteristic preferences expressed in the final generation. Our conclusions are therefore focused on the associations between colors and shapes expressed in the final populations, which is the result of testing and retesting the preferential looking that eliminates the effects from onscreen position or other concurrently presented color/shape combinations. It should be emphasized again that these experiments show that the GDEA in a multi-dimensional solution space have the potential to rapidly identify robust individual aesthetic preferences. This method of evaluating several stimuli in a single presentation exploits the ability of participants to perform multi-dimensional comparisons quickly which has a direct impact on their eye-movements, allowing the relative fitness of multiple stimuli to be evaluated simultaneously. A typical evolutionary run with 10 generations and 2 stimulus presentations per generation would require 20 presentations. In the current experiment this was sufficient to explore a “solution space” representing between 9 (3 colors, 3 shapes) and 168 (7 colors, 3 shapes, 8 orientations) different stimulus configurations. When testing the same set of stimuli in a two-alternative forced choice experiment with 10 repetitions (to get a preference measure), the number of required stimulus presentations grows approximately with the square of the number of stimuli to be compared with each other, from 9 × 8 × 10 = 720 to 168 × 167 × 10 = 280,560, illustrating how “economical” an EA can be when exploring large solution spaces (cf. Holmes and Zanker, 2008). The Building Block Hypothesis (Goldberg and Holland, 1988) goes someway to explaining how an EA performs multidimensional searches so efficiently, because they effectively test interactions across all the dimensions simultaneously with each phenotype, rather than preferential looking paradigms, including 2 AFC methods, that typically limit the number of dimensions being varied between each pair of stimuli to 1. This results in a localisation of the region in the solution space that attracts strongest interest much faster than would be possible using manual selection or sequential presentation in a two alternative forced choice (2AFC) paradigm combined with a more conventional means of varying the stimuli such as the interleaved staircase (Cornsweet, 1962).

The key benefits of GDEA—the fast sampling of a huge stimulus space, and the flexibility in testing participants without the need for them to know or understand the experimental question—makes this an attractive methodology to be explored in many different contexts, such as testing human infants or even animal studies, where description of a task to the participant is simply not possible. Furthermore, it clearly lends itself to be applied to the experimental investigation of aesthetics, which has been targeted in the present study with a well known question from the Bauhaus arts school. Because verbal instructions are needed to accompany the GDEA or in *post-hoc* controls to differentiate between aesthetic choices and unspecific responses, for instance based on stimulus saliency, the method obviously is restricted to investigate preference as such in infant or animal studies.

Fechner's (1860)methods of choice, use and production have been used with mixed results to study the question of preferred associations between shapes and colors. Kandinsky's (1923) own attempt used the method of production by asking students to color in three particular shapes. A more recent attempt to reproduce his results with a wider range of shapes and colors (Albertazzi et al., 2013), used the method of choice (task: “choose a color from the circle that you see as the one most naturally related to the shape”) and led to some distinct preferences that, however, were only partially consistent with Kandinsky's claims. Another study, using an “Implicit Association Test,” which can also be categorized as a method of choice, did not produce any significant preferences (Makin and Wuerger, 2013). As we know form another experimental study of aesthetic preference, investigating bias toward the golden ratio, different results can result from using methods of choice or production, respectively (Green, 1995). Most importantly, choice method seems to be particularly vulnerable to the range of choices presented with a tendency to cluster around the center of the range. The GDEA represents a new methodology for exploring such questions, as it combines the methods of choice and production: phenotypes are produced from other phenotypes which have been previously selected by participant's choices—by removing any high-level demand for making an aesthetic decision or carefully considered action it opens an opportunity to immediately access observer's preferences. In particular, the chances of a participant encountering their preferred phenotype are often relatively small and so it must be produced through the recombination of other members of the population. Most interestingly, the only strong association observed form in our experiments is a preference for yellow triangle, which resonates with the suggestion by Albertazzi et al. (2013) that the “warmth” and degree of “natural lightness” of hues are related to particular shape.

In our previous work, we always maintained a one-to-one relationship between the phenotype (the visual stimulus) and the chromosome (the genetic representation of the stimulus): each chromosome exactly defined a unique phenotype and each phenotype could only be represented by one chromosome. In conditions C1/C2 of the present set of experiments, as well as condition D1/D2 which are described in detail in Holmes (2010), we attempted to introduce a rotation gene which changed this genotype-phenotype relationship to become one-to-many because of the rotation symmetry properties of some shapes—for example, the rotation gene could contain any value and it had no effect on the phenotype for circles. In fact, the difference in perceivable changes as a result of genetic changes is an important consideration when using the GDEA. By introducing inequalities in the number of distinct genetic representations for a single phenotype biases are introduced in the fitness estimation unless steps are taken to recognize that different genetic representations are effectively identical from the participant's perspective. This is because the looking preferences of the participant in one-to-one mapping can be directly attributed to the individual chromosome, whereas in a many-to-one mapping, the fitness scores need somehow to be combined from similarly appearing phenotypes and attributed to all related chromosomes. One option here would be to introduce some kind of template match or image recognition component to the algorithm as part of the fitness estimation process. An alternative approach would be to represent the features which introduce the one-to-many relationships within the chromosome, rotational symmetry in this case. This way the relationship between fitness and rotational symmetry could be explored independently of the shape it is associated with.

It is important to be aware of the potential for a particular task specification to affect the behavior of the participant. In our current experiments participants were asked to look for their favorite circle, square or triangle. The explicit statement of the shape to be searched for was simply to ensure an equal number of trials were completed by each participant in the mixed shape conditions. However, asking participants to search for their favorite triangle, for example, in the mixed shape conditions b and d, immediately informed them that the other shapes were not of interest, potentially causing them to be regarded as distracters rather than be evaluated as other shapes which might be more appealing in the color currently being associated with a triangle, for example yellow. This is an example of where the use of a specific task instruction for experimental control potentially diminishes the power of the GDEA approach to experimental aesthetics which samples the entire solution space allowing the participants to freely explore the different phenotypes resulting from different gene interactions. Here the exploration was directed using a question of shape preference, but could equally have been performed using a color preference task in which participants were instructed to look for their favorite red shape, for example, and thus selecting the shape which looks best in red. The mixed color conditions would result in highly salient distracters which could easily be eliminated, suggesting a better task for exploring aesthetic preference would have been simply to look for the favorite shape (unspecified) in all conditions and use non-parametric methods to analyse the data. This relationship between the task and the genome is important particularly when the task becomes one of free-viewing since the genome must not re-introduce the biases removed by the unguided task.

In the current experiments, we simply used the ratio of cumulative fixation time (gaze positions that remained for more than 100 ms with a given region with less than ±0.25° of movement) to presentation time to determine the fitness score for each individual target in a given display. Such a method is susceptible to salience effects, attracting gaze toward the most obviously visible object in a scene, rather than being especially sensitive to attaching particular labels to object that are associated with evaluation behavior (which, for instance could lead to re-visits individual items). In subsequent experiments (Holmes and Zanker, 2012) a more specific oculo-motor signature has been further developed to look at the full time-course of fixations. However, the results in the current study suggest that salience alone was not driving the responses since if that were to be the case yellow would have always driven preference, raising the question is why this preference is so much stronger for the triangle than for the other shapes. Therefore, our experiments provide evidence of individual color-shape correspondence underlying the preferences observed here, rather than simply being driven by salience, color, or luminance effects.

Notwithstanding such limitations, the current work has shown that there is a strong correlation between the eye-movements made during decision making, and the decisions themselves. Additional work (Holmes and Zanker, 2012) using the developed oculo-motor signature that included some tests of color-shape associations (which, however, was only exploratory and should be corroborated with a larger study and the full signature), corroborates our results based on correlations between eye-movements and conscious preference. The ability of the developed subjective fitness function to predict preference needs to be established using a new set of stimuli in experiments without the explicit preference task, in order to validate whether it can be generalized to other aesthetic evaluations, which are not guided by direct instructions to participants. Various steps toward this goal by using the extended GDEA and novel stimuli have been reported elsewhere (Holmes et al., 2010; Holmes and Zanker, 2011).

## Conclusions

Taken together and keeping the limitations of the current data in mind, our present results support a view that there is a certain degree of correspondence between color and shape in all participants, and that particular preferences are reproducible for individuals. Whilst individual combinations are not necessarily consistent with Kandinsky's reported correspondences, our findings do suggest that aesthetic preference for more complex color-shape combinations as seen in art, design, and packaging might be influenced by such associations. Extending the color palette disrupts the robust preference for yellow triangles observed in the three color condition, suggesting that to some extent the constraints of Kandinsky's original experiment biased his results, as his students were not allowed the freedom to repeat the use of a single color for multiple shapes, or use additional colors, although our small sample size does not allow us to draw a conclusion with any degree of confidence. Thus, the interpretation that, for example, triangles are most preferred when they are yellow should be treated with caution. Kandinsky believed that preferences such as those for the yellow triangle was related to the characteristics of the angles that define the shapes, and at the same time could resulted from religious iconography and was directly related to its pointing upward to the Sun and God (Kandinsky, 1923, 1926). Interestingly, changing the orientation of the triangle seemed to have no clear effect on its associated color in the single shape condition, suggesting that this preference, which also shows inter-individual differences, may have its roots elsewhere. The results do suggest that correspondence may exist between a shape and a range of colors which may well have its roots in semantic associations with the geometric shapes (Leborg, 2006).

### Conflict of interest statement

The authors declare that the research was conducted in the absence of any commercial or financial relationships that could be construed as a potential conflict of interest.
